# Atomic Force Manipulation of Single Magnetic Nanoparticles for Spin-Based Electronics

**DOI:** 10.1021/acsnano.2c08622

**Published:** 2022-10-31

**Authors:** Paul Burger, Gyanendra Singh, Christer Johansson, Carlos Moya, Gilles Bruylants, Gerhard Jakob, Alexei Kalaboukhov

**Affiliations:** † Department of Microtechnology and Nanoscience - MC2, Chalmers University of Technology, GothenburgSE-41296, Sweden; ‡ Institute of Physics, Johannes Gutenberg University Mainz, Mainz55128, Germany; ¶ The Institute of Materials Science of Barcelona (ICMAB-CSIC), Barcelona08193, Spain; § RISE Research Institutes of Sweden AB, GothenburgSE-41133, Sweden; ∥ Engineering of Molecular NanoSystems, Ecole Polytechnique de Bruxelles, Université Libre de Bruxelles, Brussels1050, Belgium

**Keywords:** magnetic nanoparticles, atomic force microscopy, nanomanipulation, Hall magnetometry, oxide heterointerfaces, LAO−STO interface

## Abstract

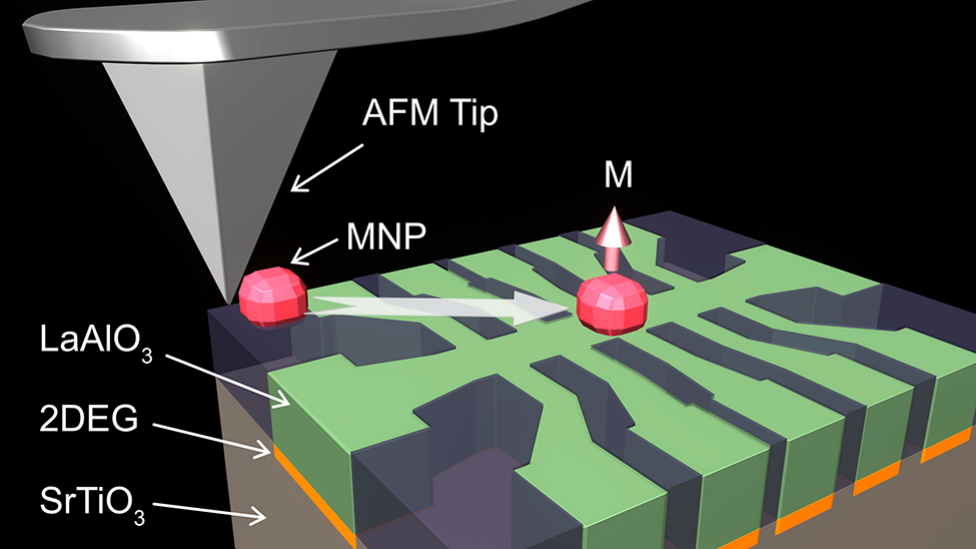

Magnetic nanoparticles (MNPs) are instrumental for fabrication of tailored nanomagnetic structures, especially where top-down lithographic patterning is not feasible. Here, we demonstrate precise and controllable manipulation of individual magnetite MNPs using the tip of an atomic force microscope. We verify our approach by placing a single MNP with a diameter of 50 nm on top of a 100 nm Hall bar fabricated in a quasi-two-dimensional electron gas (q2DEG) at the oxide interface between LaAlO_3_ and SrTiO_3_ (LAO/STO). A hysteresis loop due to the magnetic hysteresis properties of the magnetite MNPs was observed in the Hall resistance. Further, the effective coercivity of the Hall resistance hysteresis loop could be changed upon field cooling at different angles of the cooling field with respect to the measuring field. The effect is associated with the alignment of the MNP magnetic moment along the easy axis closest to the external field direction across the Verwey transition in magnetite. Our results can facilitate experimental realization of magnetic proximity devices using single MNPs and two-dimensional materials for spin-based nanoelectronics.

## Introduction

Magnetic nanoparticles (MNPs) have been widely used for decades in biomedical applications, such as contrast agents in magnetic immunoassays, imaging of tissues, drug delivery, and cancer treatment using magnetic hyperthermia.^1−4^ In addition, MNPs can be utilized to study the effects of local magnetic moments on transport properties of various nanostructures through interaction of local magnetic stray fields or magnetic proximity effects with supporting structures.^5,6^ This is especially important where traditional top-down lithographic patterning of magnetic nanostructures is impeded by surface chemical instabilities, for example in two-dimensional layered materials, such as graphene, van der Waals materials, topological insulators, and superconductors.^7^ Even complex magnetic structures may be created from several MNPs.^8,9^ For such applications, precise alignment of MNPs with nanometer precision is crucial, along with the control of their magnetic moment orientation.

The manipulation of single MNPs has been previously reported in connection with Hall magnetometry^10^ and nano-SQUID^11−15^ experiments, where weak local magnetic fields from isolated MNPs placed above the Hall bar are detected. Several approaches have been used for alignment of single iron oxide MNPs on the top of micro-Hall bars, such as lift-off through a 100 nm opening in the polymer layer,^16^ scanning tunneling microscopy-assisted chemical vapor deposition,^17^ and focused ion beam tip manipulation.^18^ However, these techniques do not allow simple and reproducible nanomanipulation or assembling of more complex structures of individual MNPs.

Atomic force microscopy (AFM) is a versatile technique that has been previously used for manipulation of metallic nanoparticles and for deliberate fabrication of nanodevices in a particle-to-particle approach with precision down to 30 nm.^19−21^ There have been also attempts to use AFM for manipulation of MNPs.^22,23^ Here, we demonstrate that AFM can be used to locate individual MNPs on a substrate and to push or drag them into desired positions over distances of several micrometers with a precision of at least 25 nm. The AFM also allows to dissect single MNPs from large clusters and position them on the surface without deteriorating their magnetic properties as verified by magnetic force microscope (MFM) measurements.

We verify our approach by performing Hall magnetometry of a single 50 nm Fe_3–*x*_O_4_ MNP using the quasi-two-dimensional electron gas (q2DEG) formed at the interface between the two wide band gap insulators SrTiO_3_ and LaAlO_3_ (LAO/STO).^24^ We have chosen the oxide q2DEG as it has several outstanding properties. High-mobility charge carriers originate from at least two energy bands,^25,26^ and they condense to a superconducting state at low temperature^27^ with coexisting intrinsic magnetic ordering.^28^ In addition, there is a very strong Rashba spin–orbit coupling (SOC)^29,30^ producing highly efficient spin-to-charge interconversion effects.^31^ These properties are potentially interesting for various applications of the LAO/STO q2DEG, including spintronics^32^ and topological superconductivity.^33,34^ On the other hand, electronic properties of the LAO/STO q2DEG are very sensitive to the presence of metallic^35,36^ and organic overlayers.^37^ In this respect, utilization of MNPs is beneficial to study local magnetic proximity with q2DEG without the need of overlayer deposition and lithographic patterning.

A single 50 nm magnetite (Fe_3–*x*_O_4_) MNP was placed using an AFM tip over a 100 nm wide Hall bar fabricated in the LAO/STO q2DEG. We observed a clear hysteresis in the Hall effect due to the presence of a single MNP above the Hall bar corresponding to the coercive field of the particle. The amplitude of the hysteresis agrees with the diffusive regime of electrical transport in the q2DEG. We also show that the magnetic moment of the single MNP can be manipulated by field-cooling through the Verwey transition, thus enabling control over the magnetic moment orientation relative to the surface plane.

## Results and Discussion

### Characterization of MNPs

#### Magnetization Measurements

Two different types of Fe_3–*x*_O_4_ MNPs have been studied: commercial multicore Bionized NanoFerrite particles (Micromod GmbH, Rostock, Germany) with a 100 nm average diameter (BNF100) and single-core particles with a diameter of 50 nm (here denoted C50) that were synthesized especially for this work.^38^

BNF-MNPs are dispersed in water and consist of about 75% magnetite and have a shell of hydroxyethyl starch. C50 MNPs were synthesized by high-temperature decomposition of Fe(III)–acetyl acetonate with decanoic acid as the capping ligand in an organic solvent, as reported in ref (38). The C50 particles are dispersed in ethanol.

**Figure 1:**
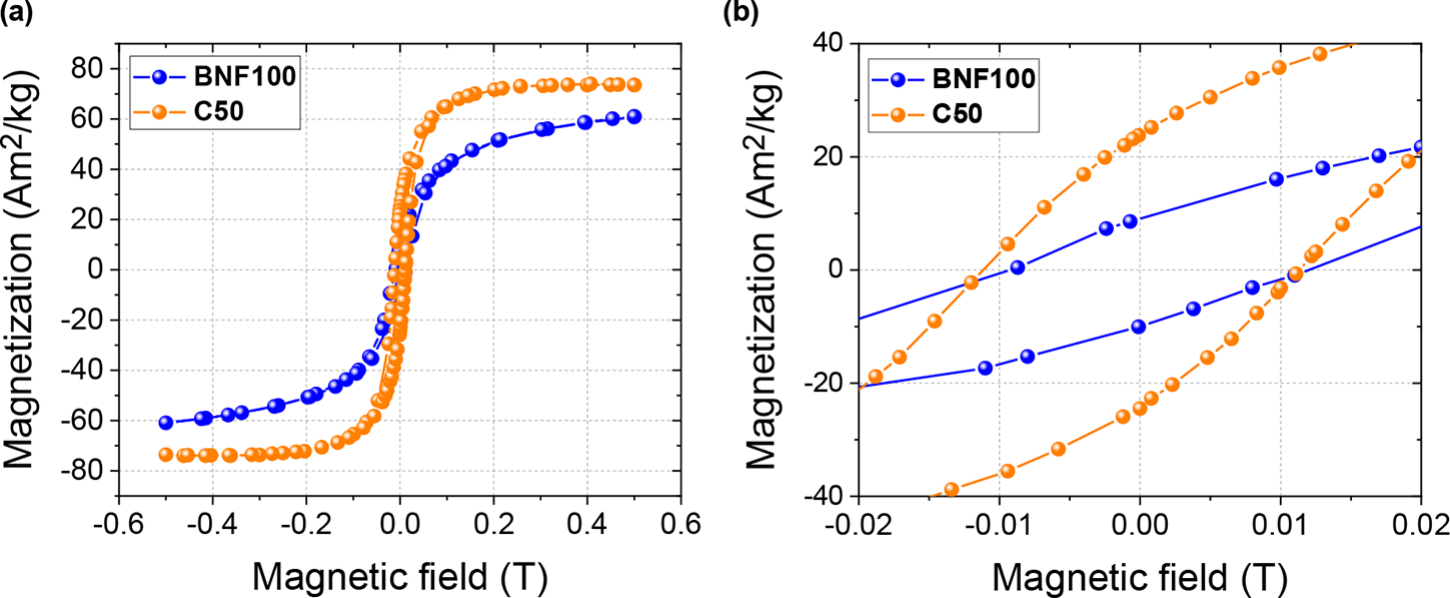
Hysteresis loops of MNP ensembles. (a) Collective hysteresis loop of two MNP systems measured with a vibrating sample magnetometer at *T* = 300 K. (b) Low-field part of the hysteresis loops showing a difference in coercive field. The measurements were performed on an ensemble of immobilized MNPs. The magnetization values were normalized to the total mass of the sample.

Measured *M–H* curves of ensembles of both types of MNPs are shown in Figure 1a. The magnetization values were normalized to the mass of each sample. The hysteresis loop for C50 MNPs is much steeper, with higher saturation magnetization, *M*_s_, at 500 mT and more than twice the magnetic remanence, *M*_r_, as compared to the BNF100 MNPs. This is a consequence of the single-crystal, single-domain structure of the C50 particles, which is also evident from the TEM measurements (see Supporting Information Figure 1). During the measurements, *M*_r_ slowly decreased with time due to slow magnetic relaxation after the MNP system had been magnetically saturated. The determined value for the saturation magnetization of C50, *M*_s_ = 73 Am^2^/kg, corresponds to about 80% of the bulk value of magnetite, *M*_s,bulk_ = 92 Am^2^/kg, at room temperature.^39^ The estimated values of the remanent magnetic moment of individual BNF100 and C50 MNPs are 2.7 × 10^–17^ and 3.1 × 10^–17^ Am^2^, correspondingly. It turns out that the remanent magnetic moment of the smaller C50 particles is estimated to be higher than that of the larger BNF100 particles. Although the nominal volume of the BNF100 MNP is higher, the magnetite mass of a single BNF100 particle is only twice the mass of the C50 MNP due to the presence of the starch shell. Also, since the BNF100 particles contain multiple magnetic cores with random individual magnetic moment orientations, their total magnetic moment is a vector sum of moments of individual domains^40^ resulting in a lower remanent effective magnetic moment than a single-domain particle with equivalent magnetite mass. At the same time, the saturation magnetic moment of BNF100 MNP is twice higher than that for C50 MNP due to the larger mass and the fact that all magnetic moments in the BNF100 particles are aligned by the magnetic field.

#### MFM Imaging

**Figure 2:**
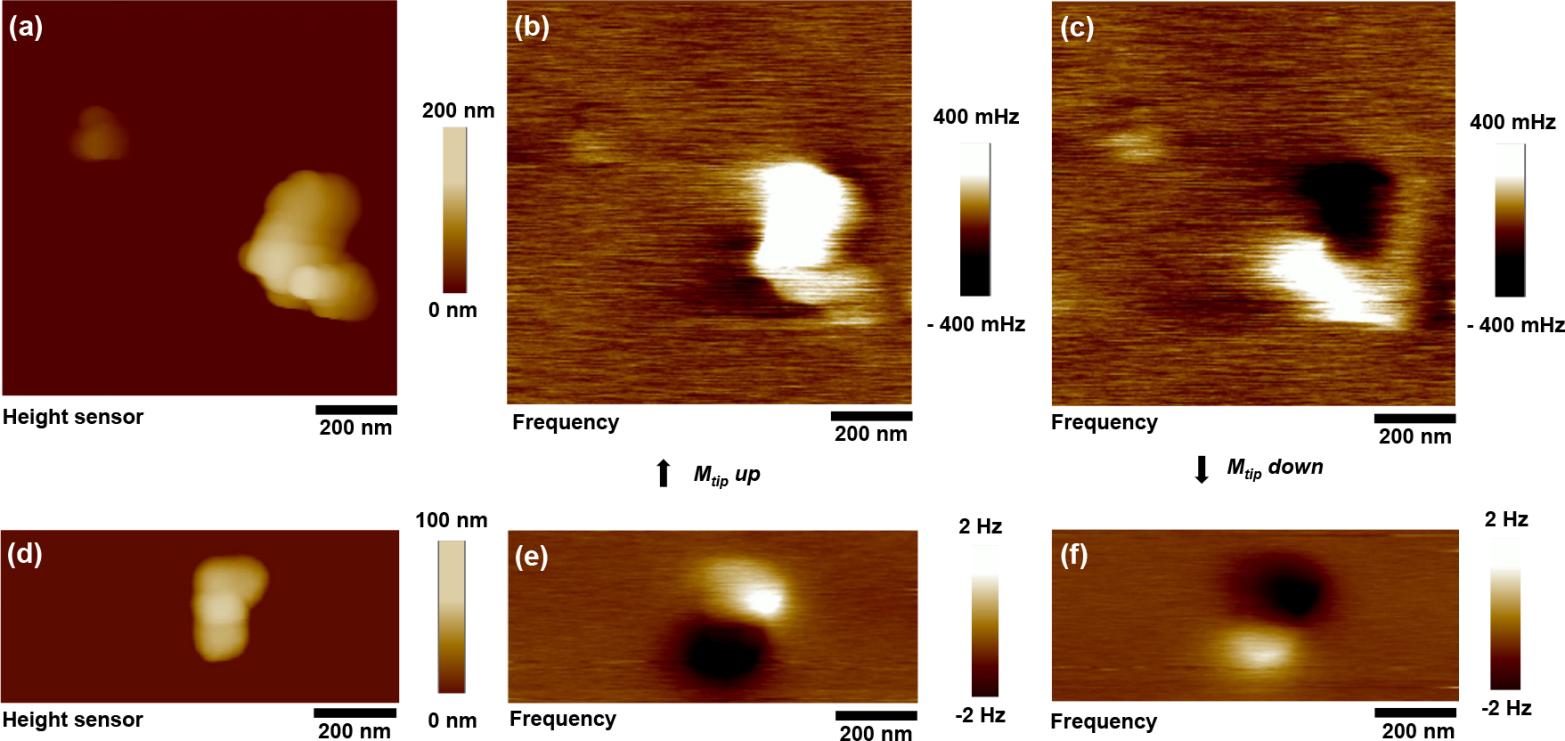
Magnetic force microscopy. Tapping mode AFM topography and MFM images of the BNF100 and C50 MNPs. (a, d) AFM topography images of the BNF100 and C50 MNPs, correspondingly. (b, c and e, f) MFM frequency contrast images of the same MNPs with magnetic moment of the tip aligned downward and upward, correspondingly. The magnetic moment alignment of the AFM tip was performed using a permanent magnet placed in proximity before each measurement.

Figure 2a,d show AFM topography images of isolated BNF100 and a cluster of two C50 MNPs, correspondingly. One can clearly see the multicore structure of the BNF100 MNP with a lateral size around 80–120 nm. The C50 MNPs appear to be square-shaped with an average size of 50 nm, as also evidenced by the transmission electron microscope (TEM) images shown in Supporting Information Figure 1. The MFM images of the same MNPs are shown in Figure 2c,d and Figure 2e,f correspondingly. For both types of MNPs, the frequency contrast in the MFM images is inverted for opposite directions of the magnetic moment of the MFM tip. From the shape of the MFM contrast, one can obtain information about the orientation of the magnetic moment of the magnetic nanoparticle (for details, see the Supporting Information). Also, the C50 MNPs appear to show stronger MFM contrast as compared with BNF100 particles. The frequency contrast in the MFM signal is proportional to ,^41^ where  is the magnetic moment of a single MNP and *d* is the particle diameter and accounts for the distance from the top of the particle plus lift height *z* to the effective magnetic moment of the tip, emerging from coating, amplitude set point, and other geometry, and *δ* the rest of the distance to the effective tip magnetic moment.^42^ Due to the smaller distance to the effective tip magnetic moment and higher remanent magnetization as discussed in the previous section, the overall MFM contrast of C50 MNPs is expected to be higher.

### AFM Nanomanipulation

The deposition of BNF100 MNPs always results in several isolated MNPs (at least one per 100 μm^2^) found between the larger clusters. More details of MNP deposition are provided in the Supporting Information. The C50 MNPs are more prone to agglomeration. They tend to collect in small, chain-like clusters, probably induced by the well-defined cubic shape of C50 MNPs. Using the tip of the AFM, isolated MNPs can be extracted from such larger clusters; see Supporting Information Figure 2 for details of the extraction process. It may take several attempts until a particle is extracted.

The manipulation is performed by instructing the AFM tip to follow a defined path at a fixed height above the surface without feedback. As the exact distance between the tip and the surface is not well-defined in tapping mode, we gradually decrease the tip height until the MNP starts moving. Usually, good results are obtained with relative heights between 30 and 50 nm and a tip with a velocity of 0.1 μm/s. It was also found that the use of electrically conductive AFM tips helps to avoid electrostatic charging effects.

**Figure 3:**
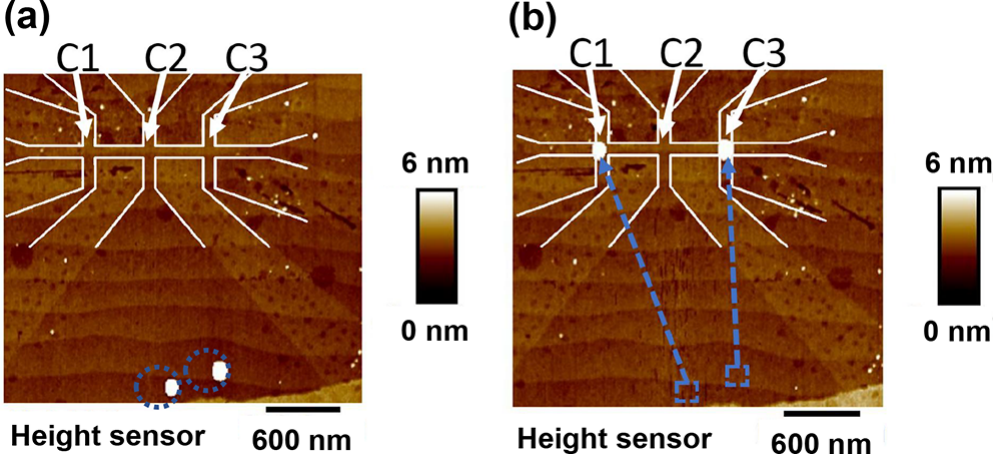
AFM nanomanipulation of magnetic nanoparticles (MNPs). (a) AFM tapping mode height image of two individual C50 MNPs before nanomanipulation of two MNPs in the bottom of the figure. (b) The same image after the AFM tip was used to move particles above the Hall bar devices with a line width of 100 nm. Dashed circles and arrows indicate nanoparticles and the path of the AFM tip, correspondingly.

Figure 3a,b show AFM topography images of two C50 MNPs before and after nanomanipulation on the top of a 100 nm wide Hall bar device fabricated in the LAO/STO interface. The MNPs can be simply moved at distances of several micrometers in one step. Since the size of the single MNP is 50 nm and that it can be positioned in the center of the 100 nm cross-bar in one step, we estimate the precision of the AFM manipulation to be at least 25 nm. The alignment precision can be further improved by repeatedly adjusting the position of the MNP in several nanomanipulation attempts and is limited mainly by the precision of the AFM tip positioning.

### Hall Effect in the Presence of MNPs

**Figure 4:**
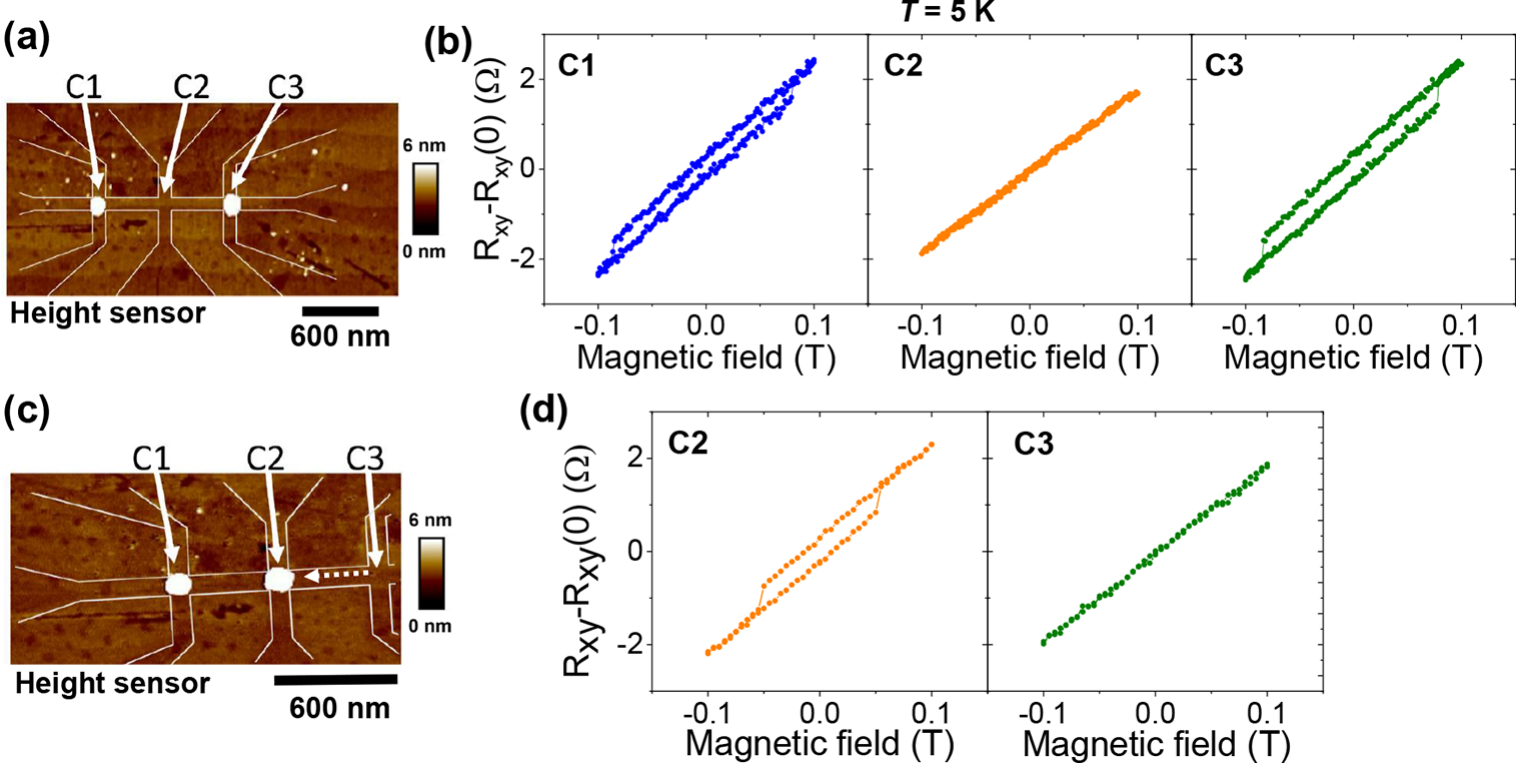
(a) Tapping mode AFM image of the nano-Hall bar structure with two C50 MNPs placed above devices C1 and C3. (b) Hall resistance *R*_*xy*_ as a function of perpendicular magnetic field. (c) Tapping mode AFM image of the same nano-Hall bar structure after one of the C50 MNPs was moved from device C3 to C2 by the AFM tip. (d) Hall resistance *R*_*xy*_ as a function of perpendicular magnetic field after movement of the MNP from C3 to C2. All electrical transport measurements were performed at *T* = 5 K using a bias current of 1 μA.

For the Hall effect measurements, we have selected C50 MNPs due to their higher remnant magnetic moment, as inferred from both magnetization and MFM measurements. Two single C50 MNPs were placed on the nano-Hall bar fabricated in the LAO/STO interface; see Figure 4. Each device had three Hall bar crosses, which allows simultaneous independent measurements of the Hall voltages using the same bias current.

The Hall resistance (*R*_*xy*_ = *V*_*yy*_/*I*_*xx*_) data are taken at *T* = 5 K after cooling in zero field and with a bias current of 1 μA. The Hall effect measured with the out-of-plane field showed the clear appearance of hysteresis for crosses C1 and C3, where the MNPs were placed above, while there was no hysteresis in cross C2, where there was no MNP placed; see Figure 4. After the measurement, the sample was warmed up to 300 K and one of the MNPs was moved from device C3 to C2 using the AFM tip; see Figure 4c. After this, the sample was cooled down again, and measurements of the Hall effect revealed that the hysteresis in the Hall effect in cross C3 has disappeared but became clearly visible for cross C2; see Figure 4d. This observation proves that the hysteresis in the Hall effects is due to the presence of the MNP.

A contribution to the Hall effect due to the stray field from the MNP is *R*_MNP_ = *R*_*H*_ × *H*_MNP_, where *R*_*H*_ = d*R*_*xy*_/d*H*_ext_ is the slope of the Hall effect in the absence of the MNP. In the case of a ballistic conduction, the Hall resistance is given by the average of the field over the cross area, *R*_*xy*_ = α⟨*H*⟩/*ne*, where α accounts for collimation effects and *n* and *e* denotes carrier concentration and electron charge, respectively.^43^ In the diffusive regime, where the electron mean free path is smaller than the dimension of the lead, the signal is reduced since the current is flowing into the voltage leads. This results in a larger effective cross area, and the response becomes more sensitive to the position and shape of the field profile.^44^ Assuming the particle to be a magnetic dipole with an out-of-plane easy axis direction, the maximum average perpendicular component of the stray field at the surface of the q2DEG from an MNP with a remanent magnetic moment of 3 × 10^–17^ Am^2^ is about ⟨*H*⟩ ≈ 0.022 T; see the Supporting Information. Assuming the slope of the Hall effect in our Hall bar to be about *R*_*xy*_ = 22 Ω/T, this results in the expected contribution to the Hall effect of about *R*_MNP_ ≈ 1 Ω for the above slope in the ballistic regime. The maximum experimental hysteresis in resistance amounts to ≈0.5 Ω. This suggests that our Hall bar is operated in the diffusive regime. Independent measurements of the Hall effect in the absence of the MNPs in our devices yielded values for charge density and mobility of *n* = 1.5 × 10^13^ cm^2^ and μ = 1200 cm^2^/(V s), corresponding to the mean free path of about 75 nm.^45^ This agrees with a diffusive transport, as the mean free path is smaller than the width and length of the Hall bar devices.

The sensitivity of the Hall magnetometer depends on the Hall coefficient (in units of Ω/T) and the equivalent resistance noise that can be calculated from the voltage noise of the device normalized to the current bias. The LAO/STO q2DEG has a rather high carrier concentration and rather low Hall coefficient of about 22 Ω/T. Assuming the resistance of the device of about 10 kΩ, the white voltage noise should not exceed 10 nV/Hz^1/2^. For the current bias of 1 μA, this corresponds to the equivalent magnetic field sensitivity of 5 × 10^–4^ T/Hz^1/2^. This is about 2 orders of magnitude higher than in the semiconductor 2DEG Hall bar magnetometers,^18^ where both Hall coefficient and current bias are much higher. At the same time, we could measure the remanent magnetic moment of the C50 MNP of about 3 × 10^–17^ Am^2^ = 3 × 10^6^ μ_B_. Assuming the estimated sensitivity of our device, we could measure a 10 times smaller magnetic moment of about 3 × 10^5^ μ_B_ with a signal-to-noise ratio of 10. This value is comparable with the results presented in earlier experiments in refs (16) and (17).

**Figure 5:**
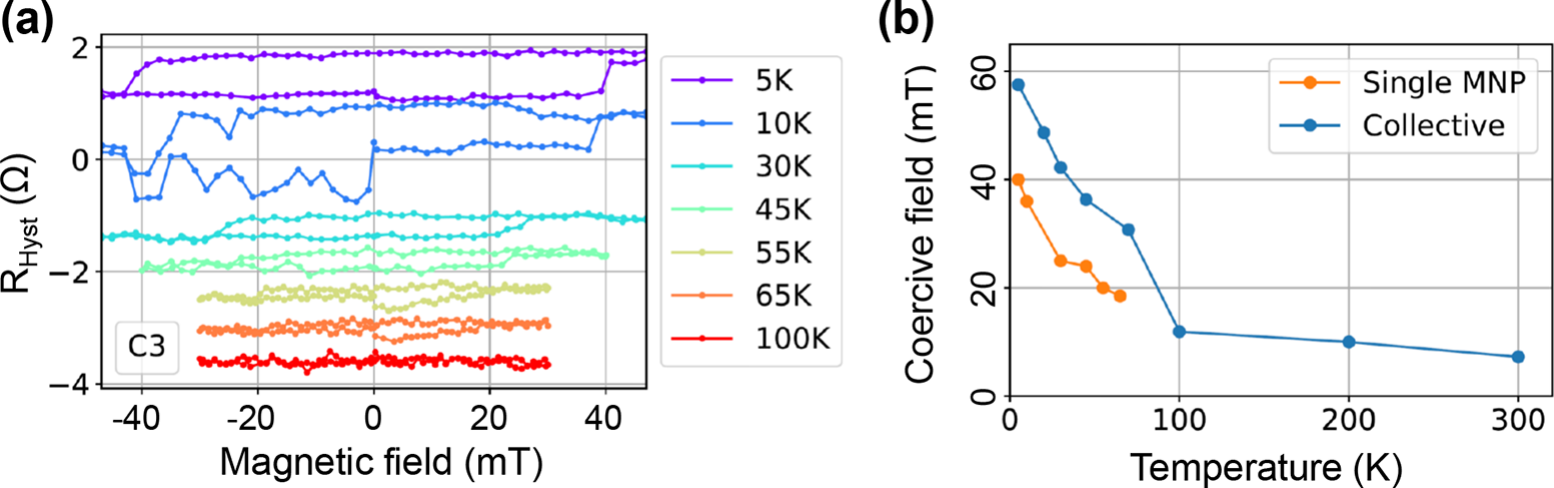
(a) Single MNP hysteresis for different temperatures measured for C50 MNP placed on a C3 Hall bar with *I*_b_ = 1 μA. The linear Hall effect was subtracted and the curves were shifted for visibility. (b). Temperature dependence of the coercive field for single and collective C50 MNPs. Hysteresis loops for single MNPs cannot be measured above 65 K due to noise. Collective MNPs show a Verwey transition around 100 K as indicated by the change of the slope in the temperature dependence of the coercive field.

**Figure 6:**
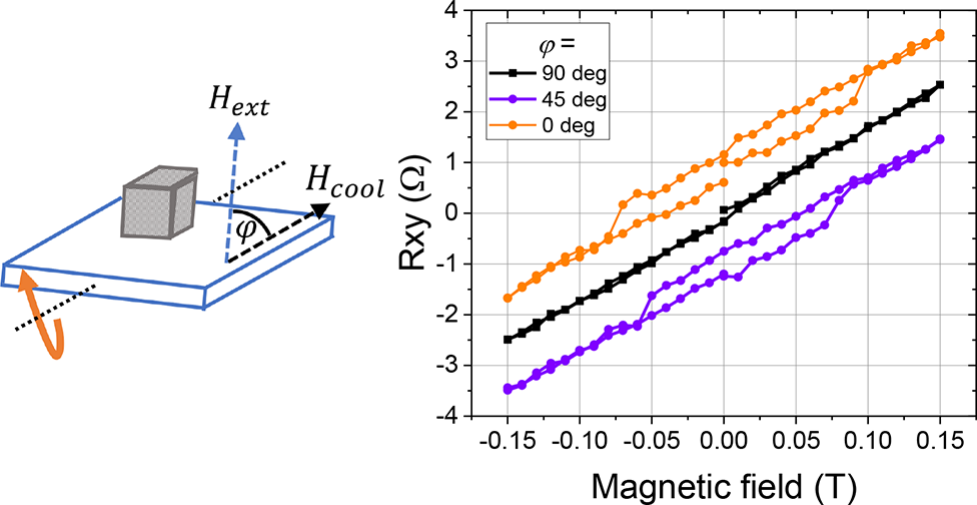
Hall effect measurements of the Hall bar device C2 as a function of angle between the magnetic field applied during cool-down (*H*_cool_) and the substrate normal. The magnetic field was applied in the temperature range from 170 to 5 K. For the Hall effect measurements, the external magnetic field, *H*_ext_, was applied in the direction perpendicular to the sample plane. The curves were shifted along the *y*-axis direction for visibility.

The hysteresis loops show no additional Barkhausen jumps, which agrees with single-domain alignment of the C50 MNPs. The coercive fields of the two MNPs in the first measurement shown in Figure 4b are comparable: *B*_c_ = 80 mT and *B*_c_ = 79 mT, for C1 and C3, respectively. After the second MNP was moved from C3 to C2, the coercive field was reduced to about 52 mT. The origin of this change is believed to be magnetite’s cubic-to-monoclinic transition at the Verwey temperature *T*_V_ ≈ 120 K, where the reordering of the lattice alters the crystalline anisotropy.^46,47^ The Verwey transition is reflected by the jump in the *B*_c_(*T*) dependence of collective MNPs; see Figure 5. The lower transition temperature may be caused by a partial oxidation of the magnetite.^48^ When no field is applied, the orientation of the monoclinic *c*-axis, associated with the easy axis, will be arbitrary. When subject to a sufficient field during the transition, the easy axis can be aligned with the cubic edge closest to the field direction.^47^Figure 6 shows Hall effect measurements of the Hall cross bar C2 that was field-cooled (FC) in *B* = 1 T at three different angles, ϕ, between the field direction and substrate surface normal. The field was applied during the cooling of the sample from *T* = 170 K to base temperature *T* = 5 K. The Hall effect was measured, as before, with ϕ = 0° (field perpendicular to sample plane). After field-cooling at ϕ = 0°, the coercivity was maximum, *B*_c_ ≈ 85 mT. However, no hysteresis (*B*_c_ = 0 T) was observed after field-cooling at ϕ = 90°. At the intermediate angle ϕ = 45°, the coercivity was *B*_c_ ≈ 50 mT. The field-cooling at ϕ = 0° therefore implies a primarily out-of-plane easy axis of the particle. Since the external field in this case is parallel to the easy axis, a maximum *B*_c_ was observed in the hysteresis loop of the Hall effect. Inversely, ϕ = 90° aligns the easy axis perpendicularly to the external field corresponding to zero coercivity.

## Conclusions

In summary, we demonstrated a reliable AFM-based procedure for isolation of MNPs from larger clusters followed by precise placement on nanodevices. We have applied this method to place a 50 nm single MNP on the 100 nm Hall bar fabricated in the q2DEG at the LAO/STO interface. The observed hysteresis in the Hall effect agrees well with the calculated average stray field produced by the MNP assuming diffusive transport in the q2DEG. Besides precise positioning of MNPs, we also show a possibility to align the magnetic moment of the MNP using field-cooling through the Verwey transition of magnetite. This may be useful for designing determined magnetic field profiles in proximity devices that operate at low temperatures. Our method may be applicable to various kinds of nanodevices, for instance spintronic nanostructures,^49^ or superconducting hybrid quantum systems to study unconventional superconducting pairing and topological effects.^50^

## Methods

Morphology and crystal structure of C50 MNPs were determined using a ThermoFisher Tecnai T20 transmission electron microscope operating at 200 kV. TEM samples were prepared by placing a drop of a diluted suspension of C50 MNPs in ethanol onto a carbon-coated Cu grid and letting it dry under a low vacuum. The particle size histogram was determined by counting at least 300 particles with ImageJ software,^51^ and the resulting histogram was fitted with a log-normal function.^52^

The crystal structure of C50 MNPs was determined by the analysis of selected area electron diffraction (SAED) images and compared with X’Pert High Score Plus patterns for bulk Fe_3_O_4_ (code: 01-086-1337). The interplanar distances (*d*-spaces) were calculated by measuring the radius between the central spot and the diffracted rings using ImageJ software. Then the reflections were indexed to (*hkl*) planes by using as a reference the patterns of bulk Fe_3_O_4_ (code: 01-086-1337).

The two types of MNPs are thermally blocked at room temperature, and their Curie temperature is around *T*_c_ ≈ 850 K.^53^ In the size range of 30–80 nm, as given for the C50 MNPs, magnetite is expected to be primarily in the single-domain state.^54^

Magnetization curves (*M*–*H*) were measured on an ensemble of diluted particles immobilized on a paper sheet in a vibrating sample magnetometer (Lakeshore Cryotronics model 7307) as a function of magnetic field at 300 K.

AFM, MFM imaging, and nanoparticle manipulation have been performed using the Bruker ICON AFM with a Nanoscope 5 controller in tapping and lift scan modes. Bruker RTESP-300 AFM probes were used for tapping mode topography measurements. For nanomanipulation, Bruker SCM-PIT V2 probes were used. For the MFM experiments, we used Bruker MESP-V2 probes (Figure 2d–f) and MESP-HR10 (Figure 2a–c) with a CoCr coating.

MNPs were deposited on the surface of the sample by pipetting the colloidal solution directly on the surface. To achieve a desirable density of particles (5–10 clusters of MNPs per 100 μm^2^, as illustrated in the Supporting Information Figure 2a) on the surface and a homogeneous distribution, MNPs were diluted to a concentration of 10 μg/mL. The selected concentration provided that it is unlikely that a chain lands directly on a device. The MNPs were dispersed by using a 2 × 5 min ultrasonic bath first at 60%, then for 5 min at 100% using 2 × 30 s vortex mixing in between these two steps prior to deposition. To avoid agglomeration, a permanent magnet was placed under the substrate to align the magnetic moments causing the particles to repel each other near the surface. The solvent was evaporated at 75 °C on a hot plate for 1 h.

Fabrication of LAO/STO nanostructures has been described in refs (45) and (55). In brief, 10 unit cell layers (4 nm) of a thin LAO film were deposited on heated, 5 × 5 mm TiO_2_-terminated STO substrates in an oxygen atmosphere by pulsed laser deposition; see ref (56) for details of the procedure. Nanodevices were fabricated by electron beam lithography followed by low-energy Ar ion beam irradiation. The width of all Hall bar lines was 100 nm. An additional advantage of this method is that the sample surface remains fairly smooth, which aids AFM manipulation.

Electrical transport and temperature dependence of M–H curves were measured in a Physical Property Measurement System at temperatures of 5–300 K and magnetic fields up to 10 T (PPMS, Quantum Design, San Diego CA, USA).

## Supporting Information Available

Structural TEM characterization of C50 MNPs, the details of the dissection process of MNPs from large clusters, estimations of the stray field from C50 MNPs, and simulations of the MFM images (PDF)

## References

[ref1] Pankhurst Q. A.; Connolly J.; Jones S.; Dobson J. Applications of Magnetic Nanoparticles in Biomedicine. J. Phys. D: Appl. Phys. 2003, 36, 167–181. 10.1088/0022-3727/36/13/201.

[ref2] Reddy L.; Arias J.; Nicolas J.; Couvreur P. Magnetic Nanoparticles: Design and Characterization, Toxicity and Biocompatibility, Pharmaceutical and Biomedical Applications. Chem. Rev. 2012, 112, 5818–5878. 10.1021/cr300068p. 23043508

[ref3] Martins P. M.; Lima A.; Ribeiro S.; Lanceros-Mendez S.; Martins P. Magnetic Nanoparticles for Biomedical Applications: From the Soul of the Earth to the Deep History of Ourselves. ACS Appl. Bio Mater. 2021, 4, 5839–5870. 10.1021/acsabm.1c00440. 35006927

[ref4] Materon E. M.; Miyazaki C. M.; Carr O.; Joshi O.; Picciani P.; Dalmaschio C.; Davis F. Magnetic Nanoparticles in Biomedical Applications: A Review. Appl. Surf. Sci. Adv. 2021, 6, 100163 10.1016/j.apsadv.2021.100163.

[ref5] Jiang C.; Ng S.; Leung C.; Pong P. Magnetically Assembled Iron Oxide Nanoparticle Coatings and Their Integration with Pseudo-Spin-Valve Thin Films. J. Mater. Chem. C 2017, 5, 252–263. 10.1039/C6TC03918A.

[ref6] Rana B.; Mondal A.; Bandyopadhyay S.; Barman A. Applications of Nanomagnets as Dynamical Systems: I. Nanotechnology 2022, 33, 062007 10.1088/1361-6528/ac2e75. 34633310

[ref7] Liu S.; Wang J.; Shao J.; Ouyang D.; Zhang W.; Liu S.; Li Y.; Zhai T. Nanopatterning Technologies of Two-Dimensional Materials for Integrated Electronic and Optoelectronic Devices. Adv. Mater. 2022, 2200734 10.1002/adma.202200734. 35501143

[ref8] Kralj S.; Makovec D. Magnetic Assembly of Superparamagnetic Iron Oxide Nanoparticle Clusters into Nanochains and Nanobundles. ACS Nano 2015, 9, 9700–9707. 10.1021/acsnano.5b02328. 26394039

[ref9] Velez C.; Torres-Dıaz I.; Maldonado-Camargo L.; Rinaldi C.; Arnold D. P. Magnetic Assembly and Cross-Linking of Nanoparticles for Releasable Magnetic Microstructures. ACS Nano 2015, 9, 10165–10172. 10.1021/acsnano.5b03783. 26364509

[ref10] Geim A. K.; Dubonos S. V.; Lok J. G. S.; Grigorieva I. V.; Maan J. C.; Theil Hansen L.; Lindelof P. E. Ballistic Hall Micromagnetometry. Appl. Phys. Lett. 1997, 71, 2379–2381. 10.1063/1.120034.

[ref11] Vohralik P. F.; Lam S. K. H. NanoSQUID Detection of Magnetization From Ferritin Nanoparticles. Supercond. Sci. Technol. 2009, 22, 064007 10.1088/0953-2048/22/6/064007.

[ref12] Hao L.; Cox D.; See P.; Gallop J.; Kazakova O. Magnetic Nanoparticle Detection Using Nano-SQUID Sensors. J. Phys. D: Appl. Phys. 2010, 43, 474004 10.1088/0022-3727/43/47/474004.

[ref13] Martínez-Pérez M. J.; Koelle D. NanoSQUIDs: Basics and Recent Advances. Phys. Sci. Rev. 2017, 2, 20175001.

[ref14] Martínez-Pérez M. J.; Müller B.; Schwebius D.; Korinski D.; Kleiner R.; Sesé J.; Koelle D. NanoSQUID Magnetometry of Individual Cobalt Nanoparticles Grown by Focused Electron Beam Induced Deposition. Supercond. Sci. Technol. 2017, 30, 024003 10.1088/0953-2048/30/2/024003.

[ref15] Martínez-Pérez M. J.; Müller B.; Lin J.; Rodriguez L. A.; Snoeck E.; Kleiner R.; Sesé J.; Koelle D. Magnetic Vortex Nucleation and Annihilation in Bi-Stable Ultra-Small Ferromagnetic Particles. Nanoscale 2020, 12, 2587–2595. 10.1039/C9NR08557B. 31939948

[ref16] Theil Kuhn L.; Geim A.; Lok J.; Hedegård P.; Ylänen K.; Jensen J.; Johnson E.; Lindelof P. Magnetisation of Isolated Single Crystalline Fe-nanoparticles Measured by a Ballistic Hall Micro-Magnetometer. Eur. Phys. J. D 2000, 10, 259–263. 10.1007/s100530050547.

[ref17] Li Y.; Xiong P.; von Molnar S.; Wirth S.; Ohno Y.; Ohno H. Hall Magnetometry on a Single Iron Nanoparticle. Appl. Phys. Lett. 2002, 80, 4644–4646. 10.1063/1.1487921.

[ref18] Kazakova O.; Panchal V.; Gallop J.; See P.; Cox D. C.; Spasova M.; Cohen L. F. Ultrasmall Particle Detection Using a Submicron Hall Sensor. J. Appl. Phys. 2010, 107, 09E708 10.1063/1.3360584.

[ref19] Junno T.; Deppert K.; Montelius L.; Samuelson L. Controlled Manipulation of Nanoparticles With an Atomic Force Microscope. Appl. Phys. Lett. 1995, 66, 3627–3629. 10.1063/1.113809.

[ref20] Kim S.; Shafiei F.; Ratchford D.; Li X. Controlled AFM Manipulation of Small Nanoparticles and Assembly of Hybrid Nanostructures. Nanotechnology 2011, 22, 115301 10.1088/0957-4484/22/11/115301. 21301077

[ref21] Martin M.; Roschier L.; Hakonen P.; Parts U.; Paalanen M.; Schleicher B.; Kauppinen E. I. Manipulation of Ag Nanoparticles Utilizing Noncontact Atomic Force Microscopy. Appl. Phys. Lett. 1998, 73, 1505–1507. 10.1063/1.122187.

[ref22] Schleicher B.; Tapper U.; Kauppinen E. I.; Martin M.; Roschier L.; Paalanen M.; Wernsdorfer W.; Benoit A. Magnetization Reversal Measurements of Size Selected Iron Oxide Particles Produced via an Aerosol Route. Appl. Organomet. Chem. 1998, 12, 315–320. 10.1002/(SICI)1099-0739(199805)12:5<315::AID-AOC723>3.0.CO;2-6.

[ref23] Pakes C. I.; George D. P.; Ramelow S.; Cimmino A.; Jamieson D. N.; Prawer S. Manipulation of Single Magnetic Protein Particles Using Atomic Force Microscopy. J. Magn. Magn. Mater. 2004, 272–276, e1231–e1233. 10.1016/j.jmmm.2003.12.299.

[ref24] Ohtomo A.; Hwang H. A High-Mobility Electron Gas at the LaAlO_3_/SrTiO_3_ Heterointerface. Nature 2004, 427, 423–426. 10.1038/nature02308. 14749825

[ref25] Joshua A.; Pecker S.; Ruhman J.; Altman E.; Ilani S. A Universal Critical Density Underlying the Physics of Electrons at the LaAlO_3_/SrTiO_3_ Interface. Nat. Commun. 2012, 3, 1129 10.1038/ncomms2116. 23072799

[ref26] Smink A. E. M.; de Boer J. C.; Stehno M. P.; Brinkman A.; van der Wiel W. G.; Hilgenkamp H. Gate-Tunable Band Structure of the LaAlO_3_-SrTiO_3_ Interface. Phys. Rev. Lett. 2017, 118, 106401 10.1103/PhysRevLett.118.106401. 28339281

[ref27] Reyren N.; Thiel S.; Caviglia A. D.; Fitting Kourkoutis L.; Hammerl G.; Richter C.; Schneider C. W.; Kopp T.; Rüetschi A.-S.; Jaccard D.; Gabay M.; Muller D. A.; Triscone J.-M.; Mannhart J. Superconducting Interfaces Between Insulating Oxides. Science 2007, 317, 1196–1199. 10.1126/science.1146006. 17673621

[ref28] Bert J. A.; Kalisky B.; Bell C.; Kim M.; Hikita Y.; Hwang H. Y.; Moler K. A. Direct Imaging of the Coexistence of Ferromagnetism and Superconductivity at the LaAlO_3_/SrTiO_3_ Interface. Nature Phys. 2011, 7, 767–771. 10.1038/nphys2079.

[ref29] Caviglia A. D.; Gabay M.; Gariglio S.; Reyren N.; Cancellieri C.; Triscone J.-M. Tunable Rashba Spin-Orbit Interaction at Oxide Interfaces. Phys. Rev. Lett. 2010, 104, 126803 10.1103/PhysRevLett.104.126803. 20366557

[ref30] Ben Shalom M.; Sachs M.; Rakhmilevitch D.; Palevski A.; Dagan Y. Tuning Spin-Orbit Coupling and Superconductivity at the LaAlO_3_/SrTiO_3_ Interface: A Magnetotransport Study. Phys. Rev. Lett. 2010, 104, 126802 10.1103/PhysRevLett.104.126802. 20366556

[ref31] Lesne E.; et al. Highly Efficient and Tunable Spin-to-Charge Conversion Through Rashba Coupling at Oxide Interfaces. Nat. Mater. 2016, 15, 1261–1267. 10.1038/nmat4726. 27571452

[ref32] Noël P.; Trier F.; Vicente Arche L. M.; Bréhin J.; Vaz D. C.; Garcia V.; Fusil S.; Barthélémy A.; Vila L.; Bibes M.; Attané J.-P. Non-Volatile Electric Control of Spin–Charge Conversion in a SrTiO_3_ Rashba System. Nature 2020, 580, 483–486. 10.1038/s41586-020-2197-9. 32322081

[ref33] Fidkowski L.; Jiang H.-C.; Lutchyn R. M.; Nayak C. Magnetic and Superconducting Ordering in One-Dimensional Nanostructures at the LaAlO_3_/SrTiO_3_ Interface. Phys. Rev. B 2013, 87, 014436 10.1103/PhysRevB.87.014436.

[ref34] Fukaya Y.; Tamura S.; Yada K.; Tanaka Y.; Gentile P.; Cuoco M. Interorbital Topological Superconductivity in Spin-Orbit Coupled Superconductors with Inversion Symmetry Breaking. Phys. Rev. B 2018, 97, 174522 10.1103/PhysRevB.97.174522.

[ref35] Pentcheva R.; Arras R.; Otte K.; Ruiz V. G.; Pickett W. E. Termination Control of Electronic Phases in Oxide Thin Films and Interfaces: LaAlO_3_/SrTiO_3_(001). Philos. Trans. R. Soc. A 2012, 370, 4904–4926. 10.1098/rsta.2012.0202. 22987035

[ref36] Lesne E.; Reyren N.; Doennig D.; Mattana R.; Jaffres H.; Cros V.; Petroff F.; Choueikani F.; Ohresser P.; Pentcheva A.; Barthelemy R.; Bibes M. Suppression of the Critical Thickness Threshold for Conductivity at the LaAlO_3_/SrTiO_3_ Interface. Nat. Commun. 2014, 5, 4291 10.1038/ncomms5291. 25000146

[ref37] Xie Y.; Bell C.; Hikita Y.; Harashima S.; Hwang H. Y. Enhancing Electron Mobility at the LaAlO_3_/SrTiO_3_ Interface by Surface Control. Adv. Mater. 2013, 25, 4735–4738. 10.1002/adma.201301798. 23852878

[ref38] Guardia P.; Perez N.; Labarta A.; Batlle X. Controlled Synthesis of Iron Oxide Nanoparticles over a Wide Size Range. Langmuir 2010, 26, 5843–5847. 10.1021/la903767e. 20000725

[ref39] Cullity B. D.; Graham C. D. Introduction to Magnetic Materials; Wiley: Hoboken, NJ, 2008.

[ref40] Ahrentorp F.; Astalan A.; Blomgren J.; Jonasson C.; Wetterskog E.; Svedlindh P.; Lak A.; Ludwig F.; van IJzendoorn L. J.; Westphal F.; Grüttner C.; Gehrke N.; Gustafsson S.; Olsson E.; Johansson C. Effective Particle Magnetic Moment of Multi-Core Particles. J. Magn. Magn. Mater. 2015, 380, 221–226. 10.1016/j.jmmm.2014.09.070.

[ref41] Krivcov A.; Junkers T.; Möbius H. Understanding Electrostatic and Magnetic Forces in Magnetic Force Microscopy: Towards Single Superparamagnetic Nanoparticle Resolution. J. Phys. Commun. 2018, 2, 075019 10.1088/2399-6528/aad3a4.

[ref42] Kazakova O.; Puttock R.; Barton C.; Corte-León H.; Jaafar M.; Neu V.; Asenjo A. Frontiers of Magnetic Force Microscopy. J. Appl. Phys. 2019, 125, 060901 10.1063/1.5050712.

[ref43] Bending S.; Oral A. Hall Effect in a Highly Inhomogeneous Magnetic Field Distribution. J. Appl. Phys. 1997, 81, 3721 10.1063/1.365494.

[ref44] Peeters F. M.; Li X. Q. Hall Magnetometer in the Ballistic Regime. Appl. Phys. Lett. 1998, 72, 572–574. 10.1063/1.120759.

[ref45] Aurino P. P.; Kalabukhov A.; Borgani R.; Haviland D. B.; Bauch T.; Lombardi F.; Claeson T.; Winkler D. Retention of Electronic Conductivity in LaAlO_3_/SrTiO_3_ Nanostructures Using a SrCuO_2_ Capping Layer. Phys. Rev. Appl. 2016, 6, 024011 10.1103/PhysRevApplied.6.024011.

[ref46] Verwey E. J. Electronic Conduction of Magnetite (Fe_3_O_4_) and its Transition Point at Low Temperatures. Nature 1939, 144, 327–328. 10.1038/144327b0.

[ref47] Kakol Z.; Król G.; Tabiś W.; Kołodziej T.; Wiśniewski A.; Stepankova H.; Chlan V.; Kusz J.; Tarnawski Z.; Kozłowski A.; Honig J. M. Easy Axis Switching in Magnetite. J. Phys.: Conf. Ser. 2011, 303, 012106 10.1088/1742-6596/303/1/012106.

[ref48] Özdemir Ö.; Dunlop D. J.; Moskowitz B. M. Changes in Remanence, Coercivity and Domain State at Low Temperature in Magnetite. Earth Planet. Sci. Lett. 2002, 194, 343–358. 10.1016/S0012-821X(01)00562-3.

[ref49] Arava H.; Barrows F.; Stiles M. D.; Petford-Long A. K. Topological Control of Magnetic Textures. Phys. Rev. B 2021, 103, L060407 10.1103/PhysRevB.103.L060407. PMC8370020 34409242

[ref50] Xiang Z.-L.; Ashhab S.; You J. Q.; Nori F. Hybrid Quantum Circuits: Superconducting Circuits Interacting With Other Quantum Systems. Rev. Mod. Phys. 2013, 85, 623–653. 10.1103/RevModPhys.85.623.

[ref51] Schneider C. A.; Rasband W. S.; Eliceiri K. W. NIH Image to ImageJ: 25 Years of Image Analysis. Nat. Methods 2012, 9, 671–675. 10.1038/nmeth.2089. 22930834 PMC5554542

[ref52] Escoda-Torroella M.; Moya C.; Rodríguez A.; Batlle X.; Labarta A. Selective Control over the Morphology and the Oxidation State of Iron Oxide Nanoparticles. Langmuir 2021, 37, 35–45. 10.1021/acs.langmuir.0c02221. 33301314

[ref53] Panda R.; Gajbhiye N.; Balaji G. Magnetic Properties of Interacting Single Domain Fe_3_O_4_ Particles. J. Alloys Compd. 2001, 326, 50–53. 10.1016/S0925-8388(01)01225-7.

[ref54] Li Q.; Kartikowati C. W.; Horie S.; Ogi T.; Iwaki T.; Okuyama K. Correlation Between Particle Size/Domain Structure and Magnetic Properties of Highly Crystalline Fe_3_O_4_ Nanoparticles. Sci. Rep. 2017, 7, 9894 10.1038/s41598-017-09897-5. 28855564 PMC5577113

[ref55] Aurino P.; Kalabukhov A.; Tuzla N.; Olsson E.; Winkler D.; Claeson T. Nano-Patterning of the Electron Gas at the LaAlO_3_/SrTiO_3_ Interface Using Low-Energy Ion Beam Irradiation. Appl. Phys. Lett. 2013, 102, 201610 10.1063/1.4807785.

[ref56] Aurino P. P.; Kalabukhov A.; Tuzla N.; Olsson E.; Klein A.; Erhart P.; Boikov Y. A.; Serenkov I. T.; Sakharov V. I.; Claeson T.; Winkler D. Reversible Metal-Insulator Transition of Ar-Irradiated LaAlO_3_/SrTiO_3_ Interfaces. Phys. Rev. B 2015, 92, 155130 10.1103/PhysRevB.92.155130.

