# Supplementary material for: Atomic Force Manipulation of Single Magnetic Nanoparticles for Spin-Based Electronics
Source: ACS Nano. 2022 Oct 31;16(11):19253–60. doi: 10.1021/acsnano.2c08622 (PMC9706809; doi:10.1021/acsnano.2c08622)
Supplement: Supplementary file 1 — nn2c08622_si_001.pdf [file nn2c08622_si_001.pdf]

Supporting Information
Atomic Force Manipulation of Single Magnetic Nanoparticles for Spin-Based Electronics

Paul Burger,[†,‡] Gyanendra Singh,[†,¶] Christer Johansson,[§,†] Carlos Moya,[‖] Gilles Bruylants,[‖] Gerhard Jakob,[‡] and Alexei Kalaboukhov[*,†]

†*Department of Microtechnology and Nanoscience - MC2, Chalmers University of Technology, Gothenburg SE-41296, Sweden*

‡*Institute of Physics, Johannes Gutenberg University Mainz, Mainz 55128, Germany*

¶*The Institute of Materials Science of Barcelona (ICMAB-CSIC), Barcelona 08193, Spain*

§*RISE Research Institutes of Sweden AB, Gothenburg SE-41258, Sweden*

‖*Engineering of Molecular NanoSystems, Ecole Polytechnique de Bruxelles, Université Libre de Bruxelles, Brussels 1050, Belgium*

E-mail: alexei.kalaboukhov@chalmers.se

Phone: +46 (0)31 7725477

Structural investigation of C50 MNPs

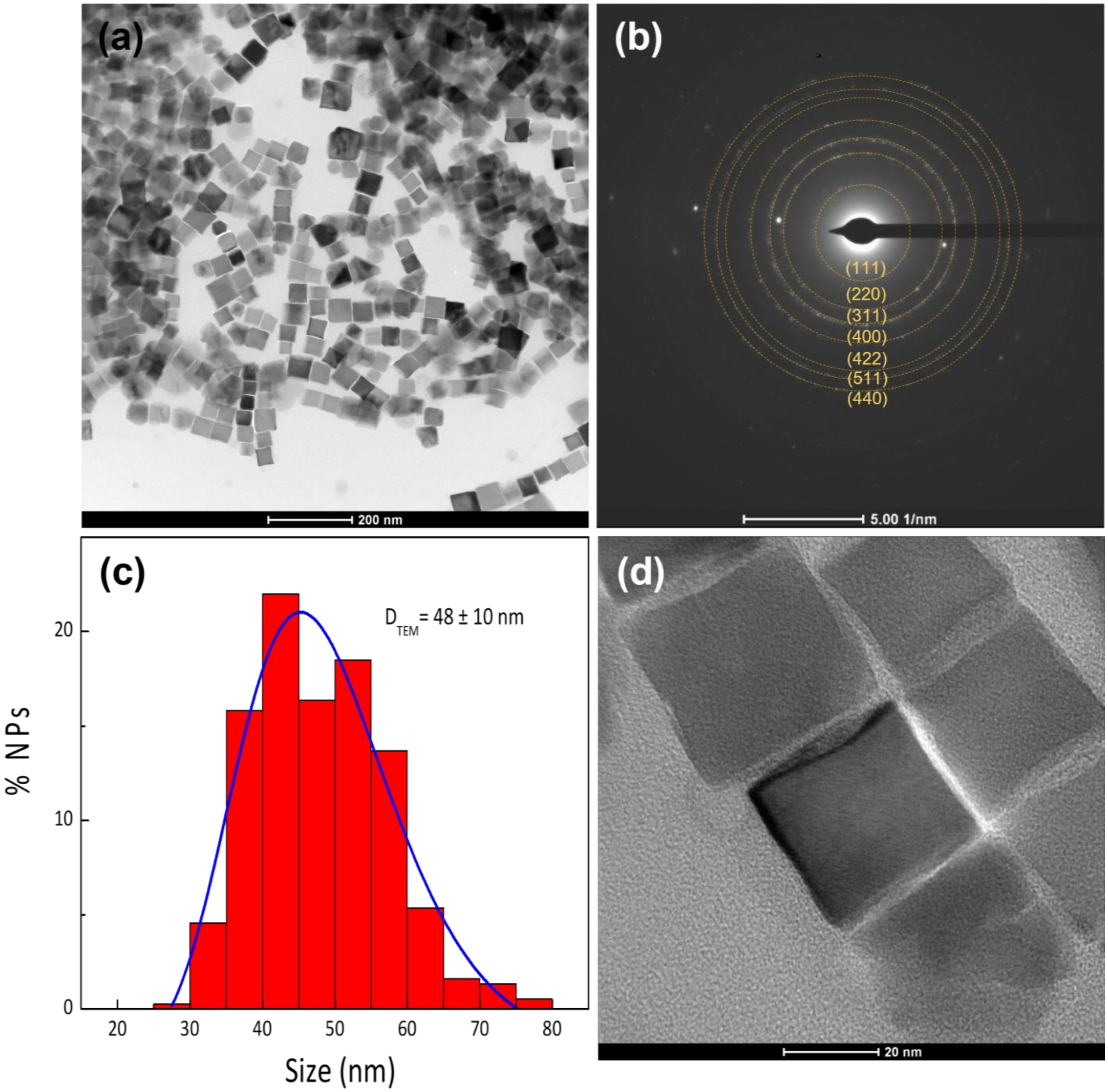

Figure 1: Structural TEM characterization of C50 MNPs. (a) Representative low magnification TEM image of a dense agglomerate MNPs. (b) SAED pattern indexed to the Fe_3O4 phase. (c) Particle size distribution obtained by TEM and fitted to a Log-normal function. (d) High Resolution-TEM image showing 6 C50 MNPs forming a self-assembled chain.

Dissection of MNP clusters

Due to the strong magnetic moment and the lack of coating, MNPs such as the C50s appear in clusters of sizes ranging from a few to dozens of particles. Fig.2 shows how such an agglomerate can be dissected using the tip of the AFM. This procedure does not yield only isolated particles but mostly smaller clusters of 2-4 particles which cannot be dissected further because the tip would just move the entire piece. We found it most effective to try to "slice" off protruding MNPs from chain-like agglomerates. With this technique we were able to extract 1-2 isolated MNPs from most clusters.

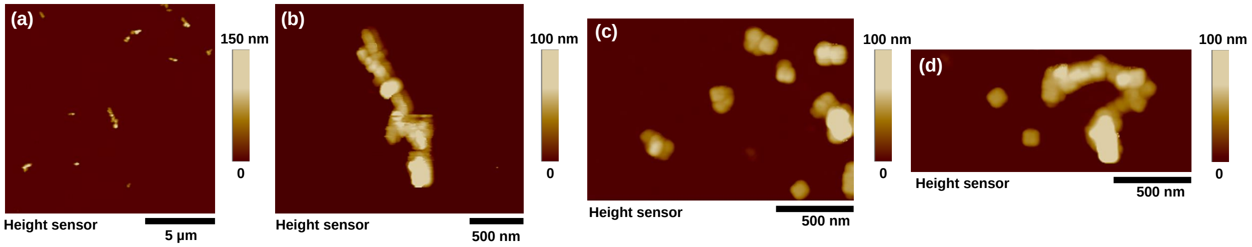

Figure 2: Cluster dissection using the tip of the AFM. (a) Overview of the area showing multiple MNP clusters after deposition. A zoom-in of the center chain is shown in (b). (c)-(d) The chain is fragmented into smaller elements, some of which are single MNPs.

Estimations of the stray field from C50 MNPs

The MNP stray field can be estimated assuming it to be originating from a single dipole levitating above the surface at height of the MNPs radius $d/2$. This assumption holds true when evaluating the field outside of a uniformly magnetized sphere. The field of such a dipole is given by the following equation:[1]

$$\mathbf{B}(\mathbf{r}) = \nabla \times \mathbf{A} = \frac{\mu_0}{4\pi}\left[\frac{3\mathbf{r}(\mathbf{m}\cdot\mathbf{r})}{r^5} - \frac{\mathbf{m}}{r^3}\right] = \frac{\mu_0}{4\pi}\frac{m}{r^3}\left[3\hat{\mathbf{r}}(\hat{\mathbf{m}}\cdot\hat{\mathbf{r}}) - \hat{\mathbf{m}}\right], \tag{1}$$

where $\mathbf{A}$ is the vector potential, $\mathbf{r} = \mathbf{r}' - \mathbf{r}_{\mathrm{mnp}}$ is the vector from MNP to a point in the q2DEG. $\mathbf{r}'$ is the vector to the point from the origin and $\mathbf{r}_{\mathrm{mnp}}$ is the position of the MNP, e.g. $\mathbf{r}^0_{\mathrm{mnp}} = \{0, 0, d/2\}$.

The thickness of the LAO layer of 4 nm and a q2DEG thickness of 10 nm is assumed. The C50 MNP has diameter $d = 50$ nm and magnetic moment $\mu = 3 \cdot 10^{-17}\mathrm{Am}^2$. The magnetic moment is directed in $\hat{\mathbf{m}} = \{\sin\Theta, 0, \cos\Theta\}$ with $\Theta = 0$ (perpendicular to the surface), For practical purposes the field is often averaged over the thickness of the q2DEG and then denoted $\langle B \rangle$.

Fig.3 shows distribution of $\langle B_\perp \rangle$ and magnetic field cross section at $y = 0$. The perpendicular component $B_\perp = \hat{\mathbf{z}} \cdot \mathbf{B}$, which is the most interesting, reaches values of above 100 mT but is highly localized. The strength of the field scales linearly with magnetic moment whereas increasing the MNP size with constant magnetic moment drastically decreases the impact on the q2DEG due to the larger distance from the effective MM in the MNP center.

Simulations of magnetic force microscopy images for MNPs

The MFM signal is proportional to the second derivative of the MNP stray field in z direction. The calculation backwards from the MFM signal to magnetic stray field and thus to magnetic moment direction is non-trivial. Here, we explicitly examine a couple of exemplary MNP - tip configurations and their corresponding MFM signal.

The magnetic forces change the resonance frequency of the tip as well as the phase of the oscillation so by recording these the contrast in an MFM image can either be expressed in terms of a phase shift $\Delta\phi$ or in terms of a (resonance-)frequency shift $\Delta\omega$. Both are proportional to the first derivative of the out-of-(sample)plane force acting on the tip.:[2,3]

$$\Delta\phi\left(\omega_0\right) \approx -\frac{\mathrm{Q}}{k}\frac{d\mathrm{F_z}}{dz} \qquad \text{and} \qquad \Delta\omega \approx -\frac{\omega_0}{2k}\frac{dF_z}{dz}, \tag{2}$$

where Q, k and ω_0 are quality factor, spring constant and resonance frequency of the

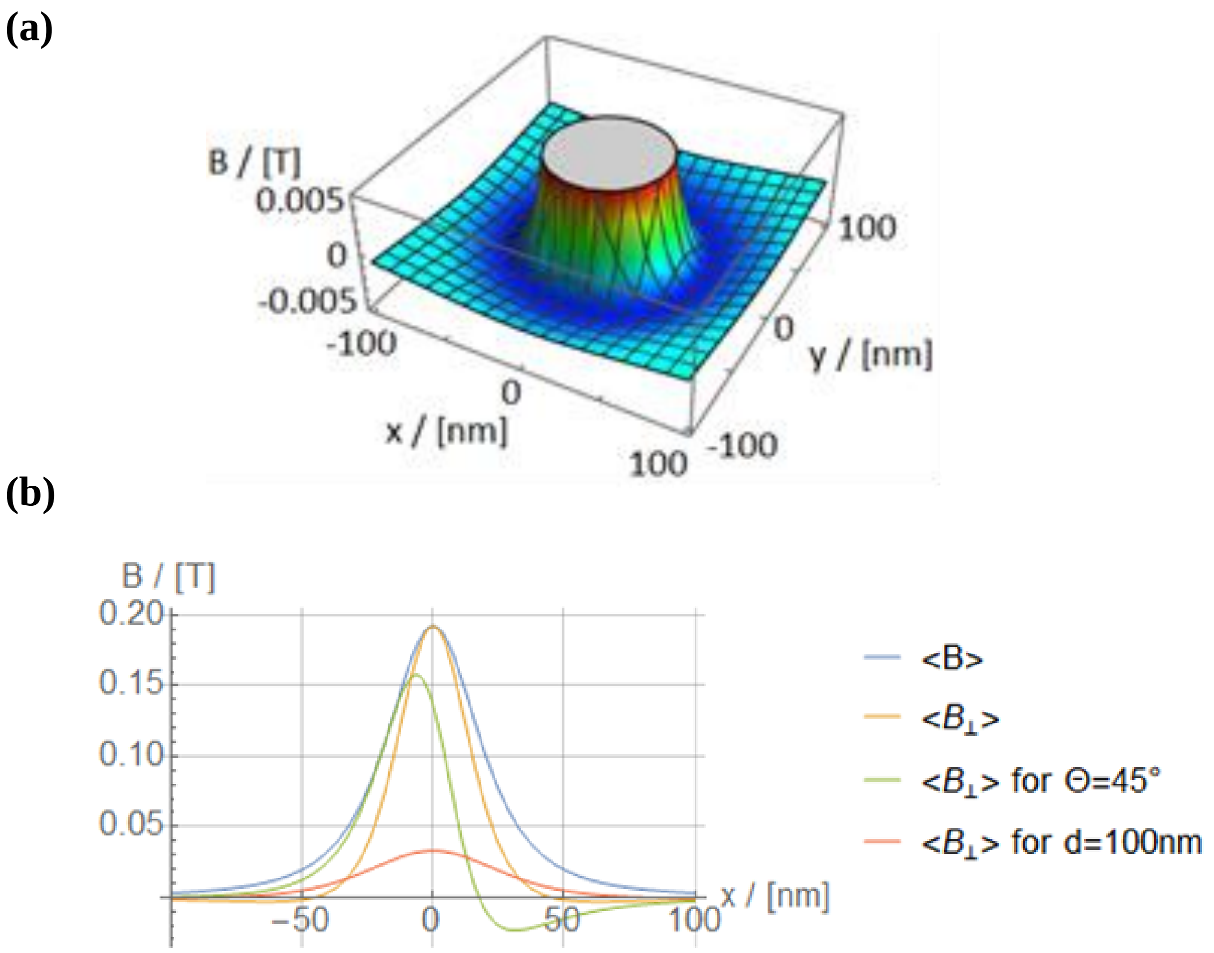

Figure 3: (a) Two-dimensional distribution of magnetic field from MNP with diameter of 50 nm and magnetic moment $\mu = 3 \cdot 10^{-17}\mathrm{Am}^2$ modelled by a dipole at $r_{MNP}(0) = 0, 0, d/2$. (b) Cross-section of magnetic field at $y = 0$ averaged over the q2DEG thickness and magnetic moment of MNP perpendicular to the surface.

tip respectively. The sign in the relations above implies that for an attractive interaction (positive force gradient), the shift in frequency or phase will be negative, which is associated with a dark contrast in the eventual image. When frequency is measured a feedback loop is applied that adjusts the frequency in order to keep the phase constant.

The MFM signal is simulated by assuming both tip and MNP to be single magnetic dipoles. In this approximation the force can be expressed analytically:

$$\begin{aligned}\mathbf{F}(\mathbf{r},\mathbf{m}_1,\mathbf{m}_2) &= \nabla(\mathbf{B}(\mathbf{r},\mathbf{m}_1)\cdot\mathbf{m}_2) \\ &= \frac{3\mu_0}{4\pi|\mathbf{r}|^4}\left(\mathbf{m}_2(\mathbf{m}_1\cdot\hat{\mathbf{r}}) + \mathbf{m}_1(\mathbf{m}_2\cdot\hat{\mathbf{r}}) + \hat{\mathbf{r}}(\mathbf{m}_1\cdot\mathbf{m}_2) - 5\hat{\mathbf{r}}(\mathbf{m}_1\cdot\hat{\mathbf{r}})(\mathbf{m}_2\cdot\hat{\mathbf{r}})\right),\end{aligned} \tag{3}$$

The derivative of the force is calculated numerically with the central difference technique for a square grid of positions. The position of the tip is given by $z_0+\delta$ above the surface, i.e. lift height plus effective distance to the MM of the tip (including e.g. tip dimensions, oscillations and coating.). The MNPs shape in x-direction, centered in the origin, is assumed to have the form $h(x) = 2re^{-(|x|/(c+r))^6}$ with particle radius r, convolution parameter c and analogously for the $y-$direction. The 3D shape of the particle is then $h(x,y) = \min\big(h(x),h(y)\big)$.

The standard parameters chosen in the simulations are: $z_0 = 40$nm, $\delta = 117$nm, $r = 30$nm, $Q = 177$, $c = 30$nm, $f_0 = 61$kHz, $k = 3$N/m and magnetic moments m_p=$3\cdot10^{-17}$Am^2, $m_\mathrm{tip} = 10^{-16}$Am^2.

Fig.4 shows simulated MFM images and MFM signal cross sections for $y = 0$ for two exemplary magnetic moment configurations of the tip/MNP system: both magnetic moments are parallel to each other, and when magnetic moment of the MNP is rotated by 45 degress relative to the tip magnetic moment. For the most common case of out-of-plane tip magnetic moment, the direction of the dark shadow is clearly related to the direction of the MNP magnetic moment. Interestingly, the MFM contrast is higher in the case of tilted- compared to exactly parallel oriented magnetic moments, see fig.4b. This is caused by the smaller

distance between the two dipoles at the edge of the particle. The consequence is a shadow in the MFM image, correlated with a dip in phase shift.

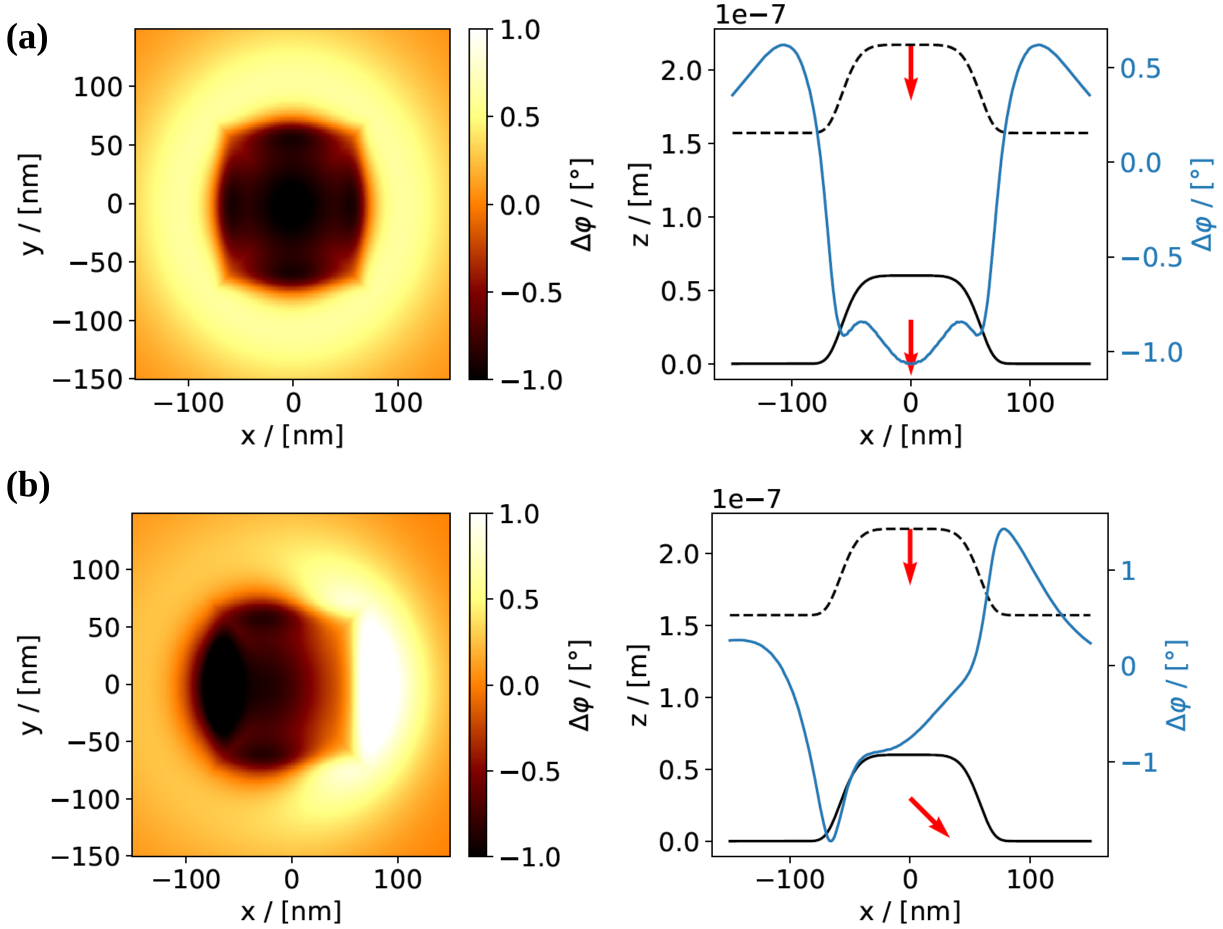

Figure 4: Simulated MFM images and their cross sections $\Delta\varphi \propto \frac{dF_z}{dz}$ (blue lines) at $y = 0$ for two exemplary magnetic moment orientations of MNP and tip: parallel (a) and tilted by 45 degrees (b). Red arrows indicate magnetic moments orientation. Black solid and dashed lines indicate the particle shape and tip scan profile, respectively.

References

(1) Sarid, D. *Scanning Force Microscopy: with Applications to Electric, Magnetic, and Atomic Forces*; Oxford University Press: New York, NY, 1994.

(2) Kazakova, O.; Puttock, R.; Barton, C.; Corte-León, H.; Jaafar, M.; Neu, V.; Asenjo, A. Frontiers of magnetic force microscopy. *Journal of Applied Physics* **2019**, *125*, 060901.

(3) Krivcov, A.; Junkers, T.; Möbius, H. Understanding electrostatic and magnetic forces in magnetic force microscopy: towards single superparamagnetic nanoparticle resolution. *Journal of Physics Communications* **2018**, *2*, 075019.
